# Bioinformatic Insights and XGBoost Identify Shared Genetics in Chronic Obstructive Pulmonary Disease and Type 2 Diabetes

**DOI:** 10.1111/crj.70057

**Published:** 2025-03-05

**Authors:** Qianqian Ji, Yaxian Meng, Xiaojie Han, Chao Yi, Xiaoliang Chen, Yiqiang Zhan

**Affiliations:** ^1^ Department of Epidemiology, School of Public Health (Shenzhen) Sun Yat‐Sen University Shenzhen Guangdong China; ^2^ Department of Chronic Disease Control Guangming Center for Disease Control and Prevention Shenzhen Guangdong China; ^3^ Guangdong Engineering Technology Research Center of Nutrition Transformation Sun Yat‐sen University Shenzhen Guangdong China; ^4^ Institute of Environmental Medicine Karolinska Institutet Stockholm Sweden

**Keywords:** chronic obstructive pulmonary disease, cross‐species validation, differentially expressed genes, machine learning, Type 2 diabetes mellitus, weighted gene co‐expression network analysis

## Abstract

**Background:**

The correlation between chronic obstructive pulmonary disease (COPD) and Type 2 diabetes mellitus (T2DM) has long been recognized, but their shared molecular underpinnings remain elusive. This study aims to uncover common genetic markers and pathways in COPD and T2DM, providing insights into their molecular crosstalk.

**Methods:**

Utilizing the Gene Expression Omnibus (GEO) database, we analyzed gene expression datasets from six COPD and five T2DM studies. A multifaceted bioinformatics approach, encompassing the limma R package, unified matrix analysis, and weighted gene co‐expression network analysis (WGCNA), was deployed to identify differentially expressed genes (DEGs) and hub genes. Functional enrichment and protein–protein interaction (PPI) analyses were conducted, followed by cross‐species validation in *Mus musculus* models. Machine learning techniques, including random forest and LASSO regression, were applied for further validation, culminating in the development of a prognostic model using XGBoost.

**Results:**

Our analysis revealed shared DEGs such as *KIF1C*, *CSTA*, *GMNN*, and *PHGDH* in both COPD and T2DM. Cross‐species comparison identified common genes including *PON1* and *CD14*, exhibiting varying expression patterns. The random forest and LASSO regression identified six critical genes, with our XGBoost model demonstrating significant predictive accuracy (AUC = 0.996 for COPD).

**Conclusions:**

This study identifies key genetic markers shared between COPD and T2DM, providing new insights into their molecular pathways. Our XGBoost model exhibited high predictive accuracy for COPD, highlighting the potential utility of these markers. These findings offer promising biomarkers for early detection and enhance our understanding of the diseases' interplay. Further validation in larger cohorts is recommended.

## Graphical abstract

This study unveils shared genetic markers between chronic obstructive pulmonary disease and Type 2 diabetes mellitus through comprehensive bioinformatic analysis and machine learning techniques. It highlights the potential of advanced algorithms like XGBoost in predicting disease outcomes, offering significant insights for early detection and personalized treatment strategies in these prevalent diseases.

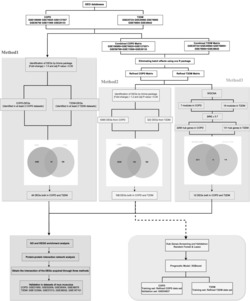

## 1 Introduction

Chronic obstructive pulmonary disease (COPD) is projected to ascend as the third leading cause of global morbidity and mortality by 2030 [1]. This complex disease arises from the interplay of genetic and environmental factors [2], notably smoking, which, despite being a major risk factor, leads to COPD in only 15%–20% of smokers [3]. This discrepancy underscores the significant role of genetic predisposition in COPD's development [4], highlighting the importance of genetic studies in understanding its pathogenesis. The global prevalence of diabetes, particularly Type 2 diabetes mellitus (T2DM), was estimated at 451 million (8.4%) in 2017 [5] and is predicted to reach 693 million (9.9%) by 2045 [6]. T2DM represents the majority of diabetes cases, indicating a rising burden of this condition [7].

The coexistence of COPD and T2DM is well documented, with individuals with COPD showing a higher prevalence of diabetes (18.7%) compared to the general population (10.5%) [8]. Furthermore, around 10% of diabetes patients are also diagnosed with COPD [9]. Although clinical and epidemiological data highlight the connection between COPD and T2DM [8, 10, 11, 12], recent genetic research has begun to uncover the molecular mechanisms that may explain this association. Studies have identified several common genetic variants and pathways that contribute to both COPD and T2DM, suggesting that these diseases may share underlying molecular mechanisms. For example, the NLRP3 inflammasome, which plays a critical role in inflammatory responses, has been implicated in both COPD exacerbations and insulin resistance in T2DM, indicating a shared pathway of inflammation [13, 14, 15]. Similarly, genetic variants in the β_2_‐adrenergic receptor gene (*ADRB2*) have been associated with both COPD severity and insulin sensitivity, further supporting the idea of a genetic overlap between these conditions [14, 16, 17]. Additionally, *PPARG*, a gene involved in glucose metabolism and inflammation, has been shown to influence both COPD pathogenesis and T2DM, highlighting the potential for shared therapeutic targets [18, 19, 20]. Despite these advances, there remains a significant gap in understanding the full extent of the shared genetic mechanisms between COPD and T2DM.

Our study aims to fill this gap by conducting a large‐scale analysis of gene expression datasets from both COPD and T2DM, utilizing data from six COPD and five T2DM studies sourced from the Gene Expression Omnibus (GEO) database. In addition to analyzing human datasets, we incorporated cross‐species validation using *Mus musculus* models to enhance the robustness and relevance of our findings. We employed the limma R package [21] and weighted gene co‐expression network analysis (WGCNA) [22] for DEG and hub gene identification, followed by functional enrichment analysis. Crucially, this study incorporates advanced machine learning techniques, including random forest [23] and LASSO regression [24], to enhance biomarker identification. Additionally, we developed a predictive model using the XGBoost algorithm [25], demonstrating substantial predictive accuracy for these diseases. This integrative bioinformatics approach sheds new light on the molecular connections between COPD and T2DM, offering potential biomarkers and insights into their shared pathophysiological pathways.

## 2 Methods

### 2.1 Data Collection and Preprocessing

We conducted a comprehensive search for gene expression datasets related to T2DM and COPD using the GEO database (http://www.ncbi.nlm.nih.gov/geo/). The search terms used were “Type 2 diabetes mellitus” and “chronic obstructive pulmonary disease,” targeting datasets that include both patient and control groups. To ensure the reliability and relevance of our analysis, we applied the following selection criteria. First, the datasets were required to include both case and control groups with a sample size of three or more for each group. We excluded datasets based on sputum samples or those involving nonstandard populations, such as individuals with HIV, to avoid confounding factors. Additionally, we ensured that control groups did not include individuals with any respiratory diseases or a family history of pulmonary conditions. Finally, only datasets that provided raw data amenable to further bioinformatic analysis were considered. After applying these criteria, six COPD studies (GSE106986, GSE76925, GSE137557, GSE56768, GSE11906, and GSE29133) and five T2DM studies (GSE25724, GSE20966, GSE76895, GSE76894, and GSE38642) were selected, encompassing 297 COPD and 553 control samples and 63 T2DM and 239 control samples, respectively (Table 1). These datasets were chosen for their comprehensive representation of gene expression alterations in these diseases. Preprocessing involved normalization of gene expression data to correct for technical variations across studies, followed by batch effect correction using the sva package [26], ensuring the analysis was based on biological rather than technical differences.

**TABLE 1:** COPD and T2DM expression profile datasets from GEO database ( *Homo sapiens* ).

Database (COPD/T2D)	Dataset ID	Continent	Platform	Number	
				Patients	Controls
COPD	GSE106986	Europe	GPL13497	5	14
COPD	GSE76925	America	GPL10558	111	40
COPD	GSE137557	America	GPL17692	8	8
COPD	GSE56768	America	GPL570	137	298
COPD	GSE11906	America	GPL570	33	190
COPD	GSE29133	Asia	GPL570	3	3
T2DM	GSE25724	Europe	GPL96	6	7
T2DM	GSE20966	America	GPL1352	10	10
T2DM	GSE76895	Europe	GPL570	19	84
T2DM	GSE76894	Europe	GPL571	19	84
T2DM	GSE38642	Europe	GPL6244	9	54

*Note:* COPD, chronic obstructive pulmonary disease; GEO, Gene Expression Omnibus; T2D, Type 2 diabetes.

### 2.2 Identification of DEGs and Hub Genes in COPD and T2DM

#### 2.2.1 Method 1: limma for DEG Identification

**FIGURE 1:**
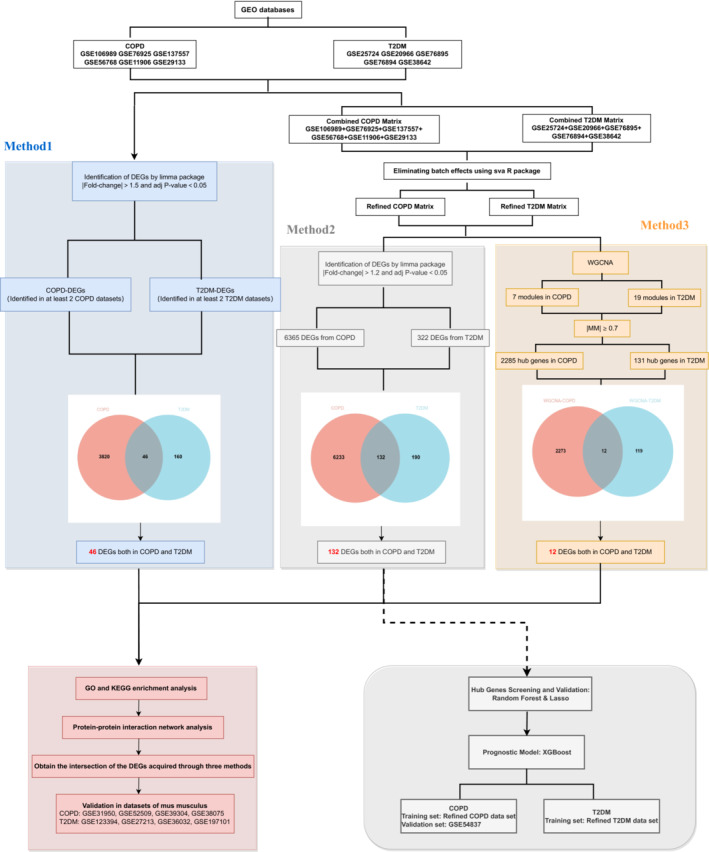
Flowchart.

We utilized the limma R package for DEG identification in COPD and T2DM datasets [21], recognized for its robust analysis capabilities, especially in complex experimental designs and small sample sizes. DEGs were defined by a |Fold‐change| > 1.5 and *p* < 0.05 in both diseases, a threshold ensuring inclusion of genes with significant expression changes. A gene was categorized as a COPD or T2DM‐associated DEG if identified in at least two of the six COPD or five T2DM studies, enhancing the DEG identification process's reliability (Figure 1).

#### 2.2.2 Method 2: Unified Matrix Analysis

Gene expression data from individual COPD and T2DM studies were integrated into unified matrices for each disease. This approach allowed comprehensive analysis across studies, enhancing the potential for relevant DEG detection. Using limma [21], genes with a |Fold‐change| > 1.2 and *p* < 0.05 were considered differentially expressed in these combined datasets (Figure 1).

#### 2.2.3 Method 3: WGCNA for Module Detection

We employed WGCNA to identify gene modules and hub genes in the combined COPD and T2DM matrices [22]. This systems biology method elucidates gene correlation patterns across microarray samples. We applied a soft thresholding power β (24 for COPD, *R* ^2^ > 0.85; 6 for T2DM, *R* ^2^ > 0.85) to achieve a scale‐free network. Modules with a minimum size of 30 genes were identified, and genes with an |MM| > 0.7 within these modules were designated as hub genes (Figure 1).

### 2.3 Function Enrichment Analysis

The Gene Ontology (GO) is a globally standardized categorization of gene functions, which offers a dynamically updated collection of controlled terminology to thoroughly depict the characteristics of genes and gene products within an organism [27]. The Kyoto Encyclopedia of Genes and Genomes (KEGG) is a comprehensive database that systematically investigates gene functionality and establishes connections between genomic information and functional information [28]. Functional enrichment analysis was executed utilizing the R package clusterProfiler [29], and the outcomes of the enrichment analysis were succinctly presented employing the academic version of the Sangerbox platform [30]. A criterion was established by setting the *p* value to be less than 0.05.

### 2.4 Protein–Protein Interaction (PPI) Network Analysis

To examine the associations among protein‐coding genes, we established a protein–protein interaction (PPI) network through the utilization of the STRING database [31] (Version 11.5; www.string‐db.org). A minimum interaction score of 0.400 ensured high confidence in predicted interactions. Genes within this network were selected for further analysis.

### 2.5 Cross‐Species Validation

To further validate the DEGs of *Homo sapiens* obtained from the above dataset, we further included COPD and T2DM datasets of *M. musculus* for validation analysis. This analysis aimed to assess the conservation of gene expression changes and identify genes potentially implicated in both human and mouse models of COPD and T2DM. Four COPD and four T2DM studies from GEO (including GSE31950, GSE52509, GSE39304, GSE38075, GSE123394, GSE27213, GSE36032, and GSE197101) were used (Table S2), and DEGs were identified using Method 1 as for the human datasets. The identified DEGs in the mouse models were then compared with those found in the human datasets to assess the cross‐species conservation of gene expression changes.

### 2.6 Machine Learning for Hub Gene Screening and Validation

Expanding on our initial identification of DEGs and hub genes in COPD and T2DM, we focused our analysis on the most abundantly identified genes across the three methods. To refine and validate these genes, we employed random forest [23] and LASSO regression [24, 32], suitable for high‐dimensional data. The random forest model was trained on 70% of the dataset with 500 trees, focusing on mean decrease accuracy for assessing gene importance. This analysis was conducted using the randomForest R package [33]. Concurrently, LASSO regression, with a binomial family model and cross‐validation, was applied to identify significant genes while minimizing overfitting. LASSO regression was implemented using the glmnet R package [32], with cross‐validation determining the optimal lambda value. The intersected results from both methods yielded a refined list of pivotal genes, which were then evaluated through receiver operating characteristic (ROC) curve analysis to quantify their diagnostic accuracy using area under the curve (AUC) values [34].

### 2.7 Development of a Prognostic Model Based on Hub Genes

Transitioning from gene validation to practical application, we constructed a prognostic model using the XGBoost algorithm [25], focusing on the refined hub genes identified for COPD and T2DM. For COPD, the model was trained using the comprehensive dataset obtained from Method 2 and validated against an independent COPD dataset, GSE56341, to ensure robustness and applicability. The XGBoost model was implemented using the xgboost R package [25], with a learning rate of 0.03, a maximum depth of 5, and 150 boosting rounds. Hyperparameters were optimized through a grid search combined with cross‐validation to enhance model performance. In the case of T2DM, although a suitable independent dataset for validation was not available, we employed similar rigorous parameter tuning techniques to improve the model's reliability and reproducibility. The final model was developed through a rigorous process of hyperparameter tuning, including grid search and cross‐validation, which optimized parameters such as learning rate, maximum depth, and the number of boosting rounds. This tuning process was critical to enhance the model's reliability, particularly in the absence of an independent validation dataset for T2DM. Features were ensured to be numeric, and missing values were imputed to maintain data integrity. The effectiveness and reliability of the prognostic model were assessed using ROC and precision–recall curves, considering AUC as the performance metric [34].

### 2.8 Software Application

All analyses were performed using R software 4.1.3 (R Core Team, Vienna, Austria). The Venn diagrams and the visualization of the enrichment analysis findings utilized in this study were carried out using the academic version of the Sangerbox platform [30].

## 3 Result

### 3.1 The DEGs/Hub Genes Identified by Three Methods in Studies of *H. sapiens*

**FIGURE 2:**
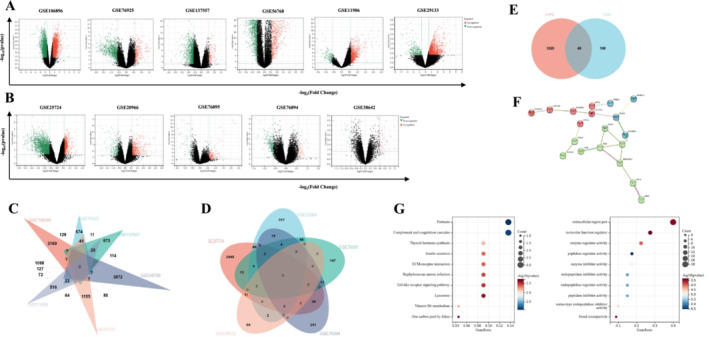
Identification and analyses of DEGs in COPD and T2DM by Method 1. (A,B) The volcano plots illustrate the differential gene expressions in six COPD (A) and five T2DM (B) datasets. The negative log10‐transformed *p* values (Y axis) are plotted against the average log2 fold changes (X axis) in gene expressions. Identified DEGs are shown in red (Fold‐change > 1.5) and green (Fold‐change < −1.5). The *p* value cutoff is < 0.05. (C,D) Venn diagrams show intersected DEGs among six COPD (C) and five T2DM (D) datasets. The area is proportional to the number of genes. (E) Venn diagram showing the total and intersected numbers of DEGs in COPD and T2DM. (F) PPI network of shared DEGs between COPD and T2DM. (G) GO and KEGG enrichment analysis of 46 DEGs shown in (E).

Using the limma R package, we analyzed six COPD and five T2DM datasets, applying a |Fold‐change| > 1.5 and *p* < 0.05 threshold. This criterion, based on the overlap of DEGs in Method 1, led to the identification of 3866 DEGs in COPD and 206 in T2DM, with 46 DEGs common to both diseases (Table S1 and Figure 2C–E). The KEGG enrichment analysis revealed that these common genes exhibited enrichment in various biological activities, such as pertussis, complement, and coagulation cascades (Figure 2G). The outcomes of GO analysis depicted that these genes exhibited enrichment in biological processes such as extracellular region part, molecular function regulator, and enzyme regulator activity (Figure 2G).

**FIGURE 3:**
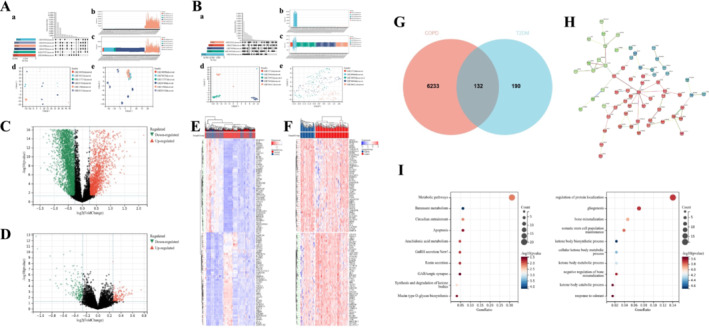
Identification and analyses of DEGs from the combined COPD and T2DM datasets by Method 2. (A,B) Normalization process based on the combined COPD/T2DM database. (a) The intersection between COPD/T2DM datasets. (b,c) Expression distribution plots for the datasets before and after normalization. (d,e) UMAP plot of the datasets before and after normalization. (C,D) The volcano plots illustrate the differential gene expressions in the combined COPD database and the combined T2DM database. (E,F) The heatmaps display the top 50 upregulated and downregulated DEGs identified from the combined COPD database and the combined T2DM database. Each row represents the intersection of genes, and each column represents one of the COPD/T2DM cases or controls. Red and blue represent upregulated and downregulated gene expression. (G) Venn diagram showing the total and intersected numbers of DEGs in COPD and T2DM. (H) PPI network of shared DEGs between COPD and T2DM. (I) GO and KEGG enrichment analysis of 132 DEGs. UMAP, Uniform Manifold Approximation and Projection.

Combining six COPD and five T2DM datasets mitigated batch effects, as evidenced in the combined matrices in Method 2 (Figure 3A,B). Applying a |Fold‐change| > 1.2 and *p* < 0.05, we found substantial variations between control groups and disease‐affected individuals, identifying 132 common DEGs (Figure 3C–G). Both the KEGG and GO analyses were utilized to examine potential functions of the common genes in COPD and T2DM. The KEGG enrichment analysis unveiled the enrichment of these genes in diverse biological activities, including metabolic pathways (Figure 3I). The findings of the GO analysis indicate that these genes exhibited enrichment in biological processes, specifically in the regulation of protein localization (Figure 3I).

**FIGURE 4:**
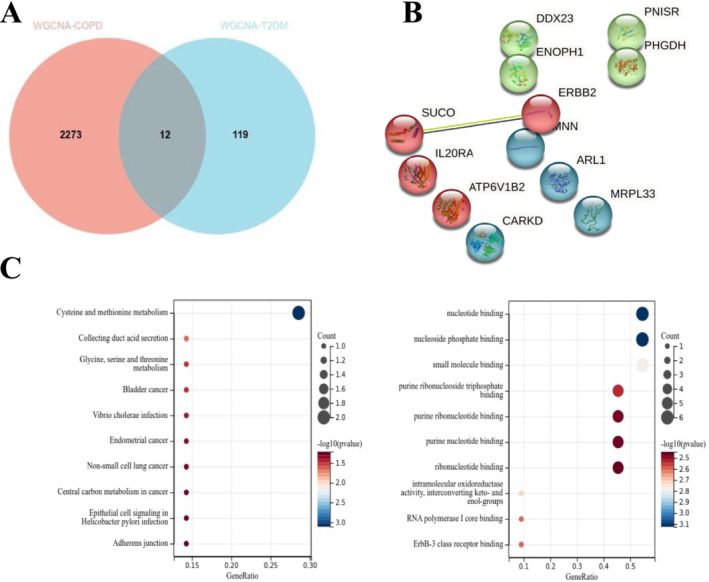
Analyses of shared hub genes between the combined COPD and T2DM datasets by Method 3. (A) Venn diagram showing the total and intersected numbers of hub genes between COPD and T2DM. (B) PPI network of shared hub genes between COPD and T2DM. (C) GO and KEGG enrichment analysis of 12 hub genes.

WGCNA of the combined COPD and T2DM database identified significant modules: black, blue, magenta, red, and royal blue for COPD and cyan, green yellow, light green, magenta, midnight blue, pink, purple, salmon, turquoise, and yellow for T2DM (Figures S1F and S2F). Utilizing a cutoff of |MM| > 0.7, we identified 2258 genes in COPD and 131 in T2DM as hub genes with significant module connectivity, including 12 common to both diseases (Figure 4A). The KEGG enrichment analysis demonstrated that the hub genes exhibited enrichment in biological processes, specifically in cysteine and methionine metabolism (Figure 4C). The findings obtained from the GO analysis indicated that these genes exhibited enrichment in biological processes including nucleotide binding, nucleoside phosphate binding, small molecule binding, purine ribonucleotide triphosphate binding, purine ribonucleotide binding, purine nucleotide binding, and ribonucleotide binding (Figure 4C).

### 3.2 KIF1C, CSTA, GMNN, and PHGDH Were Identified as the Shared DEGs in COPD and T2DM

**FIGURE 5:**
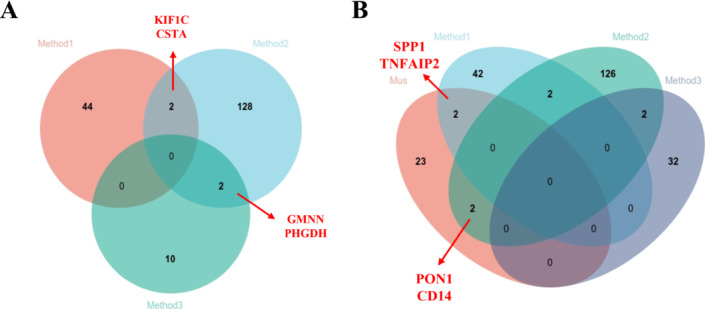
Venn diagram showing the total and intersected numbers of DEGs in COPD and T2DM. (A) Shared DEGs between three methods in *Homo sapiens* . (B) Shared DEGs between *H. sapiens* and *Mus musculus* .

The genes *KIF1C*, *CSTA*, *GMNN*, and *PHGDH* were identified as shared DEGs in both COPD and T2DM, exhibiting distinct expression patterns as confirmed by at least two of the employed methods (Figure 5A).

### 3.3 Cross‐Species DEG Analysis

Following Method 1, we identified 100 DEGs in COPD and 1852 in T2DM in *M. musculus* , with 27 common to both diseases (Figure S3A–C and Table S3). Intersecting DEGs from *M. musculus* with those from human studies revealed four shared genes: *PON1*, *CD14*, *SPP1*, and *TNFAIP2* (Figure 5B). Notably, *PON1* was downregulated in COPD and upregulated in T2DM datasets, indicating its potential role in oxidative stress‐related pathways. *CD14* was consistently upregulated in both conditions, suggesting a shared mechanism of immune dysregulation. Meanwhile, *SPP1* and *TNFAIP2* exhibited mixed expression patterns, which may reflect complex regulatory mechanisms involved in both COPD and T2DM.

### 3.4 Identification and Validation of Potential Shared hub Genes by Random Forest and LASSO

**FIGURE 6:**
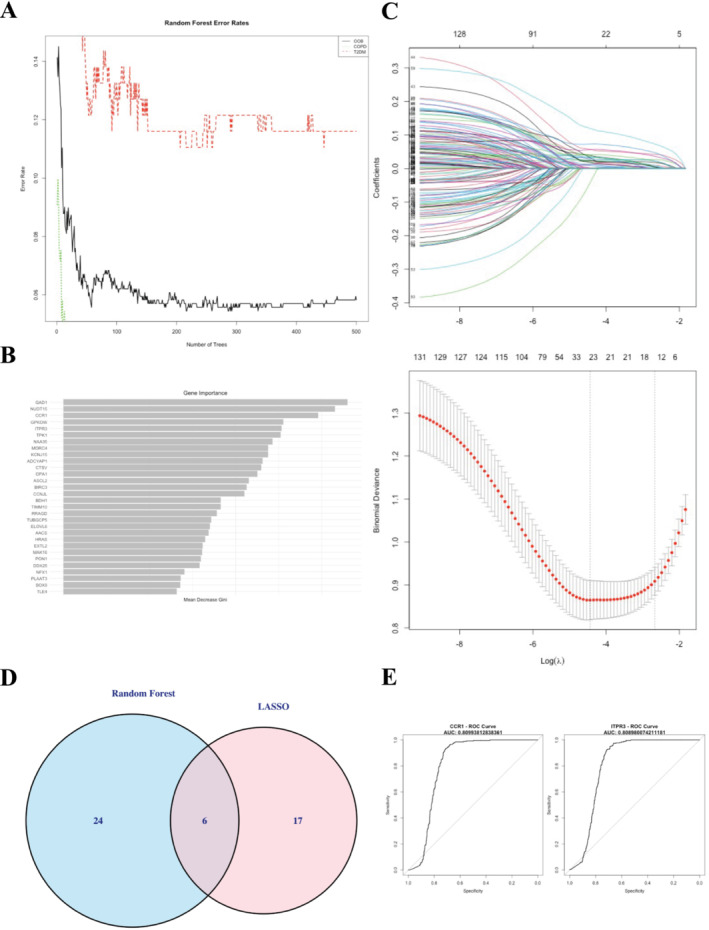
Results of random forest and LASSO. (A) Gene selection via random forest algorithm. (B) The top 30 significant genes recognized from random forest. MeanDecreaseGini showed the rank of genes in accordance with their relative importance. (C) The performance in of ten‐time cross‐verification for tuning parameter in selection least absolute shrinkage and selection operator (LASSO). (D) The intersected genes of these two algorithms were selected. (E) ROC curves of CCR1 (AUC = 0.8099, 95% CI 0.7856–0.8343) and ITPR3 (AUC = 0.8090, 95% CI 0.7847–0.8332).

Through an integrated machine learning approach, our random forest analysis delineated 18–30 genes of significance (Figure 6B), contingent on MeanDecreaseAccuracy score thresholds, whereas LASSO regression ascertained 23 genes with discernible coefficients (Figure 6C). Both analyses converged, identifying 6 pivotal genes implicated in both COPD and T2DM (Figure 6D). The random forest model's learning was demonstrated to be robust, with error rates plateauing beyond 200 trees, and LASSO regression confirmed the model's optimal complexity and accuracy at an optimal lambda, as evidenced by minimal binomial deviance. Moreover, we validated the diagnostic prognostic efficacy of each shared hub genes through ROC curve (Table S4), with *CCR1* (AUC = 0.8099) and *ITPR3* (AUC = 0.8090) having the highest AUC (Figure 6E).

### 3.5 Construction of Prognostic Model Based on XGBoost

**FIGURE 7:**
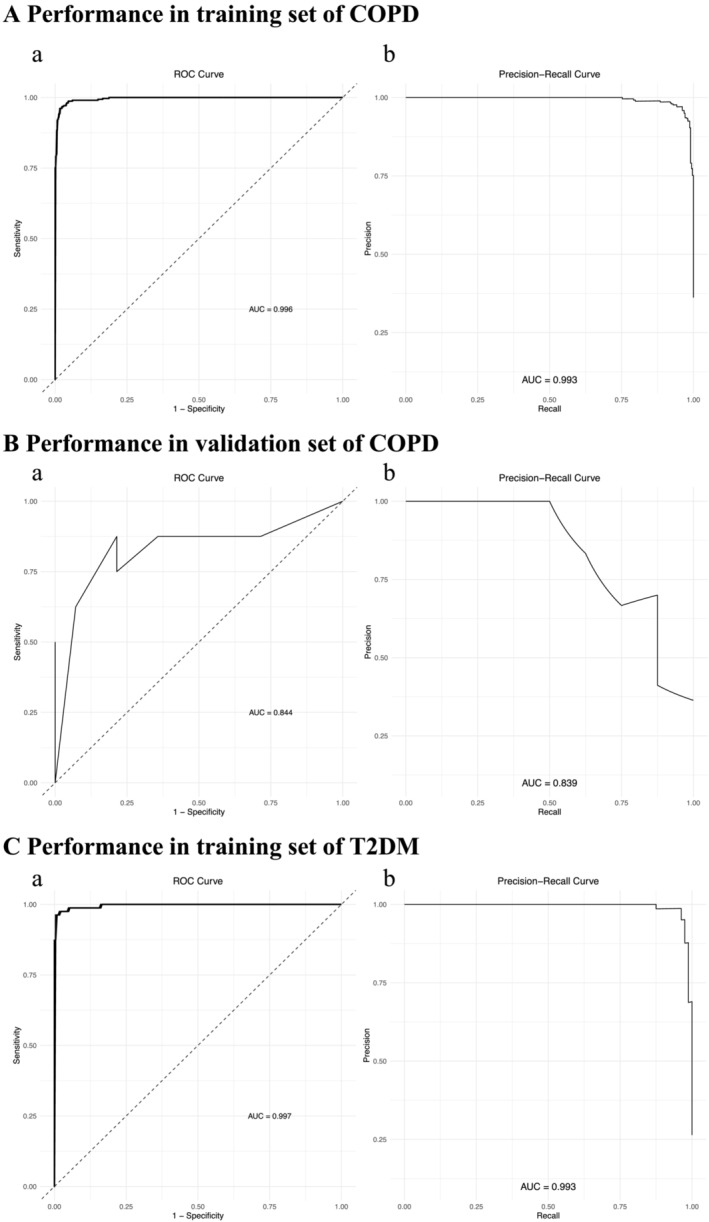
Results of XGBoost. (A) Performance in the training set (refined COPD dataset in Method 2) using XGBoost. (B) Performance in the validation set (GSE54837) using XGBoost. (C) Performance in the training set (refined T2DM dataset in Method 2) using XGBoost.

Although each shared hub gene can be employed as an auxiliary diagnostic or predictive biomarker, we prefer to develop a comprehensive prognostic model to increase the effectiveness of diagnosing or predicting diseases. Therefore, we utilized machine learning to ascertain whether these 20 hub genes can construct a comprehensive prognostic model. In this investigation, our XGBoost prognostic model, predicated on six pivotal hub genes, demonstrated remarkable predictive prowess on the COPD training set, achieving ROC and precision–recall AUCs of 0.996 and 0.993 (Figure 7A), respectively. These high metrics underscore the model's heightened sensitivity and specificity, bolstering the selected genes' status as potent discriminators and prospective biomarkers for COPD. Despite a modest diminution in the AUCs to 0.844 and 0.839 (Figure 7B) on the ROC and precision–recall curves, respectively, on the independent COPD validation set (GSE56341), the model sustained a robust generalizability. This attests to the utility of our hub gene–based prognostic model in the auxiliary diagnosis and forecasting of COPD. For T2DM, the model similarly manifested a robust performance, achieving ROC and precision–recall AUCs of 0.997 and 0.993 (Figure 7C). The absence of a T2DM validation set, due to the unavailability of an adequate high‐quality dataset, underscores the imperative for comprehensive datasets in model verification.

## 4 Discussion

Incorporating an unprecedented array of datasets, our study sheds new light on the molecular interplay between COPD and T2DM, marking a significant advancement in understanding their shared pathophysiology. The identification of key DEGs and hub genes, including *KIF1C*, *CSTA*, *GMNN*, *PHGDH*, *PON1*, *CD14*, *SPP1*, and *TNFAIP2*, underscores their pivotal roles in the etiology of both diseases. The employment of sophisticated machine learning techniques such as random forest and LASSO regression enabled the fine‐tuning of six critical genes, exhibiting substantial diagnostic accuracy, particularly *CCR1* and *ITPR3*. Furthermore, our XGBoost‐based prognostic model, built around these pivotal genes, showed remarkable predictive accuracy, especially in COPD datasets, highlighting their potential as robust biomarkers for disease management and prognosis.

Our study corroborated and expanded upon existing knowledge about key genes implicated in COPD and T2DM. *CSTA*'s role in platelet‐dependent thrombus formation, particularly its elevated expression in diabetes [35], is echoed in our findings, linking it to COPD [36, 37] and thrombotic events in COPD patients [38, 39, 40, 41, 42, 43]. This connection suggests overlapping mechanisms in thrombus formation between COPD and T2DM, opening new therapeutic avenues. *PHGDH*'s critical function in adipose tissue glucose metabolism positions it as a therapeutic target for diabetes [44, 45] with our study revealing an unexplored link to COPD. Furthermore, the differential mRNA expressions of *KIF1C* and *GMNN* in COPD and T2DM present novel insights, considering the limited understanding of these genes' biological functions.

Cross‐species comparative analysis identified additional genes, including *PON1*, *CD14*, *SPP1*, and *TNFAIP2*. Each of these genes has been implicated in crucial biological processes such as inflammation, oxidative stress, and immune response, which are central to the pathogenesis of both COPD and T2DM. *PON1*'s downregulation in COPD aligns with previous studies [46], despite contrasting findings in T2DM [47, 48], suggesting oxidative stress as a linking factor between the diseases [49, 50]. Our findings of *CD14* overexpression in COPD resonate with previous studies [51], and its altered expression post‐glucose challenge [52, 53] suggests potential mechanisms involving immune and metabolic disturbances linking both diseases [54]. The progressive upregulation of *SPP1* in COPD [55, 56] and its role as an upstream regulator in T2DM patients align with our findings [57], suggesting *SPP1*'s involvement in apoptosis, insulin resistance, and islet function regulation—pivotal pathways in the evolution of both diseases [55, 58]. Analysis of *TNFAIP2* in COPD complements prior DNA methylation studies [59, 60, 61], emphasizing its role in regulating oxidative stress and inflammation. Studies showing exacerbated inflammatory responses and oxidative stress due to *TNFAIP2* deletion align with our findings, reinforcing its potential role in diabetes and its complications [62, 63, 64, 65, 66]. However, the limited sample size in datasets like GSE36032 warrants cautious interpretation, necessitating broader studies for validation. The conservation of these genes across species underscores their fundamental roles in disease pathology and presents them as promising targets for future therapeutic strategies.

The integration of random forest and LASSO regression elevated our understanding of COPD and T2DM, with ROC curve analysis verifying the diagnostic efficacy of identified hub genes, particularly *CCR1* and *ITPR3*. *CCR1* has been recognized for its significant role in the inflammatory processes of both diseases. In COPD, *CCR1* is implicated in cigarette smoke‐induced lung inflammation, primarily mediated through the JAK/STAT/NF‐κB pathway, with its expression levels correlating with disease severity [67, 68]. This receptor's importance is further highlighted by its potential as a therapeutic target, although clinical trials with *CCR1* antagonists like AZD4818 have shown limited efficacy in improving lung function in COPD patients [69]. In T2DM, *CCR1* is involved in chronic inflammation, contributing to the progression of diabetes and its complications [70, 71]. Similarly, *ITPR3*, a gene associated with calcium signaling, has shown relevance in COPD through its regulation of intracellular Ca^2+^ release, affecting processes such as cell apoptosis and chemoresistance in lung diseases [72]. Additionally, *ITPR3* variants are associated with better survival outcomes in non–small cell lung cancer, suggesting a broader role in lung pathology [73]. In T2DM, *ITPR3* polymorphisms are linked to the disease's genetic susceptibility, particularly in autoimmune‐related pathways [74, 75]. These results not only reinforce previous research on *CCR1* [67, 68, 71] but also unveil new perspectives on *ITPR3*, broadening our understanding of their roles in disease mechanisms. The disparity in expression levels of these genes in COPD or T2DM underscores the need for a comprehensive prognostic model, hence the development of an XGBoost model. This model's high AUCs in training and validation sets demonstrate its exceptional predictive accuracy in COPD, with robust performance also observed in T2DM datasets. Although the XGBoost model showed promising results in the T2DM training set, the lack of an independent validation dataset necessitated a focused approach on hyperparameter optimization. The grid search and cross‐validation steps were crucial in ensuring the model's robustness. Nevertheless, this limitation underscores the importance of future studies utilizing extensive and independent datasets to validate and refine these biomarkers and models, ultimately ensuring their applicability in clinical settings.

Our study's inclusion of the large‐scale COPD and T2DM datasets marks a significant advance in understanding the interrelation between these diseases. It not only aids in deciphering the pathogenesis of COPD and T2DM but also enhances the utility of identified genes as biomarkers for early detection and response to treatment. The use of bioinformatic methods in our study, particularly the differential analysis across various databases using limma R package and WGCNA, showcases the capability of these methods to cross‐validate each other. To further highlight the robustness of our approach, we utilized limma both on individual datasets and on datasets after batch effect removal, alongside WGCNA for module detection. Each method brings unique strengths: limma effectively identifies DEGs in independent datasets, but its application on batch‐corrected data allows for more integrated analysis across studies. WGCNA adds another layer by identifying gene modules and hub genes. Despite their differences, these methods consistently identified similar biological pathways, reinforcing the reliability of our findings and providing a comprehensive view of the genetic architecture in COPD and T2DM. Moreover, by leveraging cross‐species validation, we have not only identified key genetic markers but also provided insights into their functional roles across different biological contexts. This dual‐species approach enhances the utility of these genes as robust biomarkers for early detection, therapeutic targeting, and monitoring of treatment response, offering new avenues for clinical intervention. Nonetheless, limitations such as the lack of a suitable T2DM validation set and the absence of in vivo or in vitro experiments to corroborate our findings highlight areas for future research.

## 5 Conclusion

In conclusion, our study offers novel insights into the shared genetic underpinnings of COPD and T2DM. The integrative approach, combining bioinformatics and machine learning, underscores the potential of these methods in biomedical research. Future studies should aim to validate these findings in larger cohorts and explore the clinical applicability of these biomarkers in disease diagnosis and management.

## Author Contributions

**Qianqian Ji:** conceptualization, methodology, software, formal analysis, writing–original draft, visualization, writing–review and editing. **Yaxian Meng:** writing–original draft, visualization. **Xiaojie Han:** writing–original draft, visualization. **Chao Yi:** writing–original draft, visualization. **Xiaoliang Chen:** conceptualization, writing–review and editing. **Yiqiang Zhan:** conceptualization, writing–review and editing.

## Ethics Statement

The authors have nothing to report.

## Consent

The authors have nothing to report.

## Conflicts of Interest

The authors declare no conflicts of interest.

## Supporting information


**Table S1** The DEGs/hub genes identified by three methods in studies of *Homo sapiens* .
**Table S2.** COPD and T2DM expression profile datasets from GEO database ( *Mus musculus* ).
**Table S3.** The DEGs identified by Method 1 in studies of *Mus musculus* .
**Table S4.** AUC of 6 hub genes.
**Figure S1** Weighted co‐expression network analysis for identification and analyses of hub genes from the combined COPD datasets (Method 3).
**Figure S2** Weighted co‐expression network analysis for identification and analyses of hub genes from the combined T2DM datasets (Method 3).
**Figure S3** Schematic plot of the combination in *Mus musculus* .

## Data Availability

The data that substantiate the conclusions of this investigation can be located within the article itself or in its accompanying supplementary material.

## References

[1] C. T. Wu , G. H. Li , C. T. Huang , et al., “Acute Exacerbation of a Chronic Obstructive Pulmonary Disease Prediction System Using Wearable Device Data, Machine Learning, and Deep Learning: Development and Cohort Study,” JMIR mHealth and uHealth 9, no. 5 (2021): e22591, 10.2196/22591. 33955840 PMC8138712

[2] Y. S. Fu , N. Kang , Y. Yu , et al., “Polyphenols, Flavonoids and Inflammasomes: The Role of Cigarette Smoke in COPD,” European Respiratory Review 31, no. 164 (2022): 220028, 10.1183/16000617.0028-2022. 35705209 PMC9648508

[3] B. Lamprecht , M. A. McBurnie , W. M. Vollmer , et al., “COPD in Never Smokers: Results From the Population‐Based Burden of Obstructive Lung Disease Study,” Chest 139, no. 4 (2011): 752–763, 10.1378/chest.10-1253. 20884729 PMC3168866

[4] L. Zhang , H. Valizadeh , I. Alipourfard , R. Bidares , L. Aghebati‐Maleki , and M. Ahmadi , “Epigenetic Modifications and Therapy in Chronic Obstructive Pulmonary Disease (COPD): An Update Review,” COPD 17, no. 3 (2020): 333–342, 10.1080/15412555.2020.1780576. 32558592

[5] G. Gudmundsson , C. S. Ulrik , T. Gislason , et al., “Long‐Term Survival in Patients Hospitalized for Chronic Obstructive Pulmonary Disease: A Prospective Observational Study in the Nordic Countries,” International Journal of Chronic Obstructive Pulmonary Disease 7 (2012): 571–576, 10.2147/COPD.S34466. 23055707 PMC3459657

[6] N. H. Cho , J. E. Shaw , S. Karuranga , et al., “IDF Diabetes Atlas: Global Estimates of Diabetes Prevalence for 2017 and Projections for 2045,” Diabetes Research and Clinical Practice 138 (2018): 271–281, 10.1016/j.diabres.2018.02.023. 29496507

[7] Y. Peng , G. C. Zhong , L. Wang , et al., “Chronic Obstructive Pulmonary Disease, Lung Function and Risk of Type 2 Diabetes: A Systematic Review and Meta‐Analysis of Cohort Studies,” BMC Pulmonary Medicine 20, no. 1 (2020): 137, 10.1186/s12890-020-1178-y. 32393205 PMC7216332

[8] M. Cazzola , G. Bettoncelli , E. Sessa , C. Cricelli , and G. Biscione , “Prevalence of Comorbidities in Patients With Chronic Obstructive Pulmonary Disease,” Respiration 80, no. 2 (2010): 112–119, 10.1159/000281880. 20134148

[9] G. E. Caughey , E. E. Roughead , A. I. Vitry , R. A. McDermott , S. Shakib , and A. L. Gilbert , “Comorbidity in the Elderly With Diabetes: Identification of Areas of Potential Treatment Conflicts,” Diabetes Research and Clinical Practice 87, no. 3 (2010): 385–393, 10.1016/j.diabres.2009.10.019. 19923032

[10] T. W. Ho , C. T. Huang , S. Y. Ruan , Y. J. Tsai , F. Lai , and C. J. Yu , “Diabetes Mellitus in Patients With Chronic Obstructive Pulmonary Disease‐The Impact on Mortality,” PLoS ONE 12, no. 4 (2017): e0175794, 10.1371/journal.pone.0175794. 28410410 PMC5391945

[11] J. S. Rana , M. A. Mittleman , J. Sheikh , et al., “Chronic Obstructive Pulmonary Disease, Asthma, and Risk of Type 2 Diabetes in Women,” Diabetes Care 27, no. 10 (2004): 2478–2484, 10.2337/diacare.27.10.2478. 15451919

[12] J. Miller , L. D. Edwards , A. Agusti , et al., “Comorbidity, Systemic Inflammation and Outcomes in the ECLIPSE Cohort,” Respiratory Medicine 107, no. 9 (2013): 1376–1384, 10.1016/j.rmed.2013.05.001. 23791463

[13] N. Nachmias , S. Langier , R. Y. Brzezinski , et al., “NLRP3 Inflammasome Activity Is Upregulated in an In‐Vitro Model of COPD Exacerbation,” PLoS ONE 14, no. 5 (2019): e0214622, 10.1371/journal.pone.0214622. 31112544 PMC6529002

[14] S. S. Park , J. L. Perez Perez , B. Perez Gandara , et al., “Mechanisms Linking COPD to Type 1 and 2 Diabetes Mellitus: Is There a Relationship Between Diabetes and COPD?,” Medicina 58, no. 8 (2022): 1030, 10.3390/medicina58081030. 36013497 PMC9415273

[15] B. Vandanmagsar , Y.‐H. Youm , A. Ravussin , et al., “The NLRP3 Inflammasome Instigates Obesity‐Induced Inflammation and Insulin Resistance,” Nature Medicine 17, no. 2 (2011): 179–188, 10.1038/nm.2279. PMC3076025 21217695

[16] S. J. Prior , A. P. Goldberg , and A. S. Ryan , “ADRB2 Haplotype Is Associated With Glucose Tolerance and Insulin Sensitivity in Obese Postmenopausal Women,” Obesity (Silver Spring) 19, no. 2 (2011): 396–401, 10.1038/oby.2010.197. 20829805 PMC3056391

[17] M. Thomsen , M. Dahl , A. Tybjaerg‐Hansen , and B. G. Nordestgaard , “β2 ‐Adrenergic Receptor Thr164IIe Polymorphism, Blood Pressure and Ischaemic Heart Disease in 66750 Individuals,” Journal of Internal Medicine 271, no. 3 (2012): 305–314, 10.1111/j.1365-2796.2011.02447.x. 21883537

[18] F. Karagiannis , S. K. Masouleh , K. Wunderling , et al., “Lipid‐Droplet Formation Drives Pathogenic Group 2 Innate Lymphoid Cells in Airway Inflammation,” Immunity 52, no. 4 (2020): 620–634.e6, 10.1016/j.immuni.2020.03.003. 32268121

[19] S. K. Solleti , D. M. Simon , S. Srisuma , et al., “Airway Epithelial Cell PPARγ Modulates Cigarette Smoke‐Induced Chemokine Expression and Emphysema Susceptibility in Mice,” American Journal of Physiology Lung Cellular and Molecular Physiology 309, no. 3 (2015): L293–L304, 10.1152/ajplung.00287.2014. 26024894 PMC4525123

[20] Y. Su , Y. Zhang , and J. Xu , “Genetic Variations in Anti‐Diabetic Drug Targets and COPD Risk: Evidence From Mendelian Randomization,” BMC Pulmonary Medicine 24, no. 1 (2024): 240, 10.1186/s12890-024-02959-1. 38750544 PMC11094874

[21] M. E. Ritchie , B. Phipson , D. Wu , et al., “limma Powers Differential Expression Analyses for RNA‐Sequencing and Microarray Studies,” Nucleic Acids Research 43, no. 7 (2015): e47, 10.1093/nar/gkv007. 25605792 PMC4402510

[22] P. Langfelder and S. Horvath , “WGCNA: An R Package for Weighted Correlation Network Analysis,” BMC Bioinformatics 9 (2008): 559, 10.1186/1471-2105-9-559. 19114008 PMC2631488

[23] L. Breiman , “Random Forests,” Machine Learning 45, no. 1 (2001): 5–32, 10.1023/A:1010933404324.

[24] R. Tibshirani , “Regression Shrinkage and Selection via the Lasso,” Journal of the Royal Statistical Society: Series B: Methodological 58, no. 1 (2018): 267–288, 10.1111/j.2517-6161.1996.tb02080.x.

[25] T. Chen and C. Guestrin , “XGBoost: A Scalable Tree Boosting System,” in *Proceedings of the 22nd ACM SIGKDD International Conference on Knowledge Discovery and Data Mining, San Francisco, California, USA*, (2016), 10.1145/2939672.2939785.

[26] J. T. Leek , W. E. Johnson , H. S. Parker , A. E. Jaffe , and J. D. Storey , “The sva Package for Removing Batch Effects and Other Unwanted Variation in High‐Throughput Experiments,” Bioinformatics 28, no. 6 (2012): 882–883, 10.1093/bioinformatics/bts034. 22257669 PMC3307112

[27] The Gene Ontology Consortium , “The Gene Ontology Resource: 20 Years and Still Going Strong,” Nucleic Acids Research 47, no. D1 (2019): D330–D338, 10.1093/nar/gky1055. 30395331 PMC6323945

[28] M. Kanehisa and S. Goto , “KEGG: Kyoto Encyclopedia of Genes and Genomes,” Nucleic Acids Research 28, no. 1 (2000): 27–30, 10.1093/nar/28.1.27. 10592173 PMC102409

[29] G. Yu , L. G. Wang , Y. Han , and Q. Y. He , “clusterProfiler: An R Package for Comparing Biological Themes Among Gene Clusters,” OMICS 16, no. 5 (2012): 284–287, 10.1089/omi.2011.0118. 22455463 PMC3339379

[30] W. Shen , Z. Song , X. Zhong , et al., “Sangerbox: A Comprehensive, Interaction‐Friendly Clinical Bioinformatics Analysis Platform. Commentary,” iMeta 1, no. 3 (2022): e36, 10.1002/imt2.36. 38868713 PMC10989974

[31] D. Szklarczyk , A. L. Gable , K. C. Nastou , et al., “The STRING Database in 2021: Customizable Protein‐Protein Networks, and Functional Characterization of User‐Uploaded Gene/Measurement Sets,” Nucleic Acids Research 49, no. D1 (2021): D605–D612, 10.1093/nar/gkaa1074. 33237311 PMC7779004

[32] S. Engebretsen and J. Bohlin , “Statistical Predictions With glmnet,” Clinical Epigenetics 11, no. 1 (2019): 123, 10.1186/s13148-019-0730-1. 31443682 PMC6708235

[33] J. Alderden , G. A. Pepper , A. Wilson , et al., “Predicting Pressure Injury in Critical Care Patients: A Machine‐Learning Model,” American Journal of Critical Care 27, no. 6 (2018): 461–468, 10.4037/ajcc2018525. 30385537 PMC6247790

[34] Q. M. Zhou , L. Zhe , R. J. Brooke , M. M. Hudson , and Y. Yuan , “A Relationship Between the Incremental Values of Area Under the ROC Curve and of Area Under the Precision‐Recall Curve,” Diagnostic and Prognostic Research 5, no. 1 (2021): 13, 10.1186/s41512-021-00102-w. 34261544 PMC8278775

[35] A. Mezzapesa , D. Bastelica , L. Crescence , et al., “Increased Levels of the Megakaryocyte and Platelet Expressed Cysteine Proteases Stefin A and Cystatin A Prevent Thrombosis,” Scientific Reports 9, no. 1 (2019): 9631, 10.1038/s41598-019-45805-9. 31270351 PMC6610149

[36] M. W. Butler , T. Fukui , J. Salit , et al., “Modulation of Cystatin A Expression in Human Airway Epithelium Related to Genotype, Smoking, COPD, and Lung Cancer,” Cancer Research 71, no. 7 (2011): 2572–2581, 10.1158/0008-5472.CAN-10-2046. 21325429 PMC3209453

[37] A. S. Kononikhin , K. Y. Fedorchenko , A. M. Ryabokon , et al., “Proteomic Analysis of Exhaled Breath Condensate for Diagnosis of Pathologies of the Respiratory System,” Biomeditsinskaya Khimiya 61, no. 6 (2015): 777–780, 10.18097/PBMC20156106777. 26716752

[38] Y. Liu , X. Meng , J. Feng , X. Zhou , and H. Zhu , “Hypereosinophilia With Concurrent Venous Thromboembolism: Clinical Features, Potential Risk Factors, and Short‐Term Outcomes in a Chinese Cohort,” Scientific Reports 10, no. 1 (2020): 8359, 10.1038/s41598-020-65128-4. 32433573 PMC7239859

[39] D. Mispelaere , J. C. Glerant , M. Audebert , A. Remond , M. A. Sevestre‐Pietri , and V. Jounieaux , “Embolie pulmonaire et formes sibilantes des decompensations de bronchopneumopathie chronique obstructive [Pulmonary Embolism and Sibilant Types of Chronic Obstructive Pulmonary Disease Decompensations],” Revue des Maladies Respiratoires 19, no. 4 (2002): 415–423. 12417857

[40] X. Shi and H. Li , “Anticoagulation Therapy in Patients With Chronic Obstructive Pulmonary Disease in the Acute Exacerbation Stage,” Experimental and Therapeutic Medicine 5, no. 5 (2013): 1367–1370, 10.3892/etm.2013.1001. 23737881 PMC3671742

[41] A. Undas , M. Jankowski , P. Kaczmarek , K. Sladek , and K. Brummel‐Ziedins , “Thrombin Generation in Chronic Obstructive Pulmonary Disease: Dependence on Plasma Factor Composition,” Thrombosis Research 128, no. 4 (2011): e24–e28, 10.1016/j.thromres.2011.05.004. 21624643 PMC3183323

[42] D. Jimenez , A. Agusti , E. Tabernero , et al., “Effect of a Pulmonary Embolism Diagnostic Strategy on Clinical Outcomes in Patients Hospitalized for COPD Exacerbation: A Randomized Clinical Trial,” Journal of the American Medical Association 326, no. 13 (2021): 1277–1285, 10.1001/jama.2021.14846. 34609451 PMC8493436

[43] O. R. Petris , E. Cojocaru , A. P. Fildan , and C. Cojocaru , “COPD and Anticoagulation Therapy: Time for a New Approach?,” International Journal of Chronic Obstructive Pulmonary Disease 16 (2021): 3429–3436, 10.2147/COPD.S340129. 34955638 PMC8694113

[44] K. Okabe , I. Usui , K. Yaku , Y. Hirabayashi , K. Tobe , and T. Nakagawa , “Deletion of PHGDH in Adipocytes Improves Glucose Intolerance in Diet‐Induced Obese Mice,” Biochemical and Biophysical Research Communications 504, no. 1 (2018): 309–314, 10.1016/j.bbrc.2018.08.180. 30180949

[45] S. Oh , Y. Cho , M. Chang , S. Park , and H. Kwon , “Metformin Decreases 2‐HG Production Through the MYC‐PHGDH Pathway in Suppressing Breast Cancer Cell Proliferation,” Metabolites 11, no. 8 (2021): 480, 10.3390/metabo11080480. 34436421 PMC8402004

[46] J. Watanabe , K. Kotani , and A. Gugliucci , “Paraoxonase 1 and Chronic Obstructive Pulmonary Disease: A Meta‐Analysis,” Antioxidants 10, no. 12 (2021): 1891, 10.3390/antiox10121891. 34942993 PMC8750165

[47] C. Wu , D. Wu , M. Lin , and Y. Zhong , “The Associations Between Paraoxonase 1 L55M/Q192R Genetic Polymorphisms and the Susceptibilities of Diabetic Macroangiopathy and Diabetic Microangiopathy: A Meta‐Analysis,” Diabetes Theraphy 9, no. 4 (2018): 1669–1688, 10.1007/s13300-018-0466-5. PMC6064588 29987647

[48] A. M. Ahmed , “Correlation of Paraoxonase‐1 With Glycated Hemoglobin and Lipid Profile Among Sudanese Diabetic Patients,” Pakistan Journal of Medical Sciences 35, no. 4 (2019): 1050–1054, 10.12669/pjms.35.4.26. 31372141 PMC6659064

[49] A. C. Maritim , R. A. Sanders , and J. B. Watkins, 3rd , “Diabetes, Oxidative Stress, and Antioxidants: A Review,” Journal of Biochemical and Molecular Toxicology 17, no. 1 (2003): 24–38, 10.1002/jbt.10058. 12616644

[50] N. Sarioglu , C. Bilen , C. Cevik , and N. Gencer , “Paraoxonase Activity and Phenotype Distribution in Patients With Chronic Obstructive Pulmonary Disease,” Eurasian Journal of Medicine 52, no. 2 (2020): 161–165, 10.5152/eurasianjmed.2019.19122. 32612424 PMC7311125

[51] S. Poliska , E. Csanky , A. Szanto , et al., “Chronic Obstructive Pulmonary Disease‐Specific Gene Expression Signatures of Alveolar Macrophages as well as Peripheral Blood Monocytes Overlap and Correlate With Lung Function,” Respiration 81, no. 6 (2011): 499–510, 10.1159/000324297. 21430361

[52] M. Koc , M. Siklova , V. Sramkova , et al., “Signs of Deregulated Gene Expression Are Present in Both CD14(+) and CD14(−) PBMC From Non‐Obese Men With Family History of T2DM,” Frontiers in Endocrinology (Lausanne) 11 (2020): 582732, 10.3389/fendo.2020.582732. PMC7917286 33658980

[53] M. A. Valtierra‐Alvarado , J. E. Castaneda‐Delgado , G. Lugo‐Villarino , et al., “Increased Frequency of CD14(+)HLA‐DR(−/Low) Cells in Type 2 Diabetes Patients With Poor Glycemic Control,” Human Immunology 83, no. 11 (2022): 789–795, 10.1016/j.humimm.2022.08.011. 36028458

[54] T. S. Kapellos , L. Bonaguro , I. Gemund , et al., “Human Monocyte Subsets and Phenotypes in Major Chronic Inflammatory Diseases,” Frontiers in Immunology 10 (2019): 2035, 10.3389/fimmu.2019.02035. 31543877 PMC6728754

[55] T. W. Miao , W. Xiao , L. Y. Du , et al., “High Expression of SPP1 in Patients With Chronic Obstructive Pulmonary Disease (COPD) Is Correlated With Increased Risk of Lung cancer,” FEBS Open Bio 11, no. 4 (2021): 1237–1249, 10.1002/2211-5463.13127. PMC8016137 33626243

[56] M. N. Ali , M. Mori , T. C. J. Mertens , et al., “Osteopontin Expression in Small Airway Epithelium in COPD Is Dependent on Differentiation and Confined to Subsets of Cells,” Scientific Reports 9, no. 1 (2019): 15566, 10.1038/s41598-019-52208-3. 31664154 PMC6820743

[57] A. K. Cheema , P. Kaur , A. Fadel , N. Younes , M. Zirie , and N. M. Rizk , “Integrated Datasets of Proteomic and Metabolomic Biomarkers to Predict Its Impacts on Comorbidities of Type 2 Diabetes Mellitus,” Diabetes & Metabolism Syndrome and Obesity 13 (2020): 2409–2431, 10.2147/DMSO.S244432. PMC7354282 32753925

[58] B. Huang , W. Wen , and S. Ye , “TSH‐SPP1/TRbeta‐TSH Positive Feedback Loop Mediates fat Deposition of Hepatocyte: Crosstalk Between Thyroid and Liver,” Frontiers in Immunology 13 (2022): 1009912, 10.3389/fimmu.2022.1009912. 36300106 PMC9589424

[59] H. Yao and I. Rahman , “Current Concepts on Oxidative/Carbonyl Stress, Inflammation and Epigenetics in Pathogenesis of Chronic Obstructive Pulmonary Disease,” Toxicology and Applied Pharmacology 254, no. 2 (2011): 72–85, 10.1016/j.taap.2009.10.022. 21296096 PMC3107364

[60] L. Cheng , J. Liu , B. Li , S. Liu , X. Li , and H. Tu , “Cigarette Smoke‐Induced Hypermethylation of the GCLC Gene Is Associated With COPD,” Chest 149, no. 2 (2016): 474–482, 10.1378/chest.14-2309. 26087411

[61] E. A. Vucic , R. Chari , K. L. Thu , et al., “DNA Methylation Is Globally Disrupted and Associated With Expression Changes in Chronic Obstructive Pulmonary Disease Small Airways,” American Journal of Respiratory Cell and Molecular Biology 50, no. 5 (2014): 912–922, 10.1165/rcmb.2013-0304OC. 24298892 PMC4068945

[62] M. Hashimoto , F. Bhuyan , M. Hiyoshi , et al., “Potential Role of the Formation of Tunneling Nanotubes in HIV‐1 Spread in Macrophages,” Journal of Immunology 196, no. 4 (2016): 1832–1841, 10.4049/jimmunol.1500845. 26773158

[63] K. Yamamoto‐Nonaka , M. Koike , K. Asanuma , et al., “Cathepsin D in Podocytes Is Important in the Pathogenesis of Proteinuria and CKD,” Journal of the American Society of Nephrology 27, no. 9 (2016): 2685–2700, 10.1681/ASN.2015040366. 26823550 PMC5004641

[64] F. Barutta , S. Kimura , K. Hase , et al., “Protective Role of the M‐Sec‐Tunneling Nanotube System in Podocytes,” Jounal of the American Society of Nephrology 32, no. 5 (2021): 1114–1130, 10.1681/ASN.2020071076. PMC8259684 33722931

[65] A. Rustom , “The Missing Link: Does Tunnelling Nanotube‐Based Supercellularity Provide a New Understanding of Chronic and Lifestyle Diseases?,” Open Biology 6, no. 6 (2016): 160057, 10.1098/rsob.160057. 27278648 PMC4929939

[66] F. Barutta , S. Bellini , S. Kimura , et al., “Protective Effect of the Tunneling Nanotube‐TNFAIP2/M‐sec System on Podocyte Autophagy in Diabetic Nephropathy,” Autophagy 19, no. 2 (2023): 505–524, 10.1080/15548627.2022.2080382. 35659195 PMC9851239

[67] K. Zhao , R. Dong , Y. Yu , et al., “Cigarette Smoke‐Induced Lung Inflammation in COPD Mediated via CCR1/JAK/STAT/NF‐κB Pathway,” Aging 12, no. 10 (2020): 9125–9138, 10.18632/aging.103180. 32463796 PMC7288948

[68] F. Wang and B. He , “CCR1 and CCR5 Expression on Inflammatory Cells Is Related to Cigarette Smoking and Chronic Obstructive Pulmonary Disease Severity,” Chinese Medical Journal 125, no. 23 (2012): 4277–4282. 23217400

[69] H. A. Kerstjens , L. Bjermer , L. Eriksson , K. Dahlström , and J. Vestbo , “Tolerability and Efficacy of Inhaled AZD4818, a CCR1 Antagonist, in Moderate to Severe COPD Patients,” Respiratory Medicine 104, no. 9 (2010): 1297–1303, 10.1016/j.rmed.2010.04.010. 20466530

[70] S. Ribeiro and R. Horuk , “The Clinical Potential of Chemokine Receptor Antagonists,” Pharmacology & Therapeutics 107, no. 1 (2005): 44–58, 10.1016/j.pharmthera.2005.01.004. 15894378

[71] M. Mraz , Z. Lacinova , J. Drapalova , et al., “The Effect of Very‐Low‐Calorie Diet on mRNA Expression of Inflammation‐Related Genes in Subcutaneous Adipose Tissue and Peripheral Monocytes of Obese Patients With Type 2 Diabetes Mellitus,” Journal of Clinical Endocrinology & Metabolism 96, no. 4 (2011): E606–E613, 10.1210/jc.2010-1858. 21289263

[72] Y. Xue , J. L. Morris , K. Yang , et al., “SMARCA4/2 Loss Inhibits Chemotherapy‐Induced Apoptosis by Restricting IP3R3‐Mediated Ca^2+^ Flux to Mitochondria,” Nature Communications 12, no. 1 (2021): 5404, 10.1038/s41467-021-25260-9. PMC8438089 34518526

[73] Y. Wu , Z. Liu , D. Tang , et al., “Potentially Functional Variants of HBEGF and ITPR3 in GnRH Signaling Pathway Genes Predict Survival of Non‐small Cell Lung cancer Patients,” Translational Research: The Journal of Laboratory and Clinical Medicine 233 (2021): 92–103, 10.1016/j.trsl.2020.12.009. 33400994 PMC8184605

[74] H.‐Q. Qu , L. Marchand , A. Szymborski , R. Grabs , and C. Polychronakos , “The Association Between Type 1 Diabetes and the ITPR3 Gene Polymorphism due to Linkage Disequilibrium With HLA Class II,” Genes and Immunity 9, no. 3 (2008): 264–266, 10.1038/gene.2008.12. 18340361

[75] Y.‐M. Chen , Q. Zhu , J. Cai , et al., “Upregulation of T Cell Receptor Signaling Pathway Components in Gestational Diabetes Mellitus Patients: Joint Analysis of mRNA and circRNA Expression Profiles,” Frontiers in Endocrinology 2021, no. 12 (2021): 774608, 10.3389/fendo.2021.774608. PMC8763273 35046894

