# Supplementary material for: Bioinformatic Insights and XGBoost Identify Shared Genetics in Chronic Obstructive Pulmonary Disease and Type 2 Diabetes
Source: Clin Respir J. 2025 Mar 5;19(3):e70057. doi: 10.1111/crj.70057 (PMC11882755; doi:10.1111/crj.70057)
Supplement: Supplementary file 1 — Table S1 The DEGs/hub genes identified by three methods in studies of Homo sapiens . Table S2. COPD and T2DM expression profile datasets from GEO database ( Mus musculus ). Table S3. The DEGs identified by Method 1 in studies of Mus musculus . Table S4. AUC of 6 hub genes. Figure S1 Weighted co‐expression network analysis for identification and analyses of hub genes from the combined COPD datasets (Method 3). Figure S2 Weighted co‐expression network analysis for identification and analyses of hub genes from the combined T2DM datasets (Method 3). Figure S3 Schematic plot of the combination in Mus musculus . [file CRJ-19-e70057-s001.docx]

**Supplementary files**

Table S1. The DEGs/hub genes identified by three methods in studies of homo sapiens

Table S2. COPD and T2DM expression profile data sets from GEO database (*mus musculus*)

Table S3. The DEGs identified by Method1 in studies of *mus musculus*

Table S4. AUC of 6 hub genes

Fig.S1 Weighted co-expression network analysis for identification and analyses of hub genes from the combined COPD datasets (Method3)

Fig.S2 Weighted co-expression network analysis for identification and analyses of hub genes from the combined T2DM datasets (Method3)

Fig.S3 Schematic plot of the combination in *mus musculus*

Table S1. The DEGs/hub genes identified by three methods in studies of homo sapiens

| **NO** | **Method1** | **Method2** | **Method3** |
| --- | --- | --- | --- |
| 1 | *FGG* | *TSN* | *ARL1* |
| 2 | *THBS2* | *NUDT15* | *ATP6V1B2* |
| 3 | *GBP3* | *CAPN7* | *DDX23* |
| 4 | *PRPH* | *OPA1* | *ENOPH1* |
| 5 | *GBP2* | *ZNF226* | *ERBB2* |
| 6 | *PPP4R1* | *NFX1* | *GMNN* |
| 7 | *NKX2-5* | *DMTN* | *IL20RA* |
| 8 | *SLC24A3* | *SCRN3* | *MRPL33* |
| 9 | *C3* | *HRAS* | *NAXD* |
| 10 | *LIPT1* | *TMEM135* | *PHGDH* |
| 11 | *ODC1* | *PCIF1* | *PNISR* |
| 12 | *KIF1C* | *RIOK2* | *SUCO* |
| 13 | *MAGEL2* | *NAA35* |  |
| 14 | *LGI2* | *SLC25A17* |  |
| 15 | *PSAT1* | *MSN* |  |
| 16 | *NSG1* | *ZFP36L2* |  |
| 17 | *CTSE* | *MRPL17* |  |
| 18 | *TNFAIP2* | *CDK8* |  |
| 19 | *CREB3L1* | *LIN7B* |  |
| 20 | *SERPINI1* | *MDFIC* |  |
| 21 | *SNX7* | *HMGN5* |  |
| 22 | *CSTA* | *COQ3* |  |
| 23 | *MICA* | *TOMM20* |  |
| 24 | *POT1* | *ZNF10* |  |
| 25 | *ZNF205* | *EIF2B3* |  |
| 26 | *SERPING1* | *HNRNPH1* |  |
| 27 | *KCNH8* | *EXTL2* |  |
| 28 | *RBP4* | *MAK16* |  |
| 29 | *CHGA* | *GPKOW* |  |
| 30 | *LY96* | *EAF2* |  |
| 31 | *GPR183* | *ZNF136* |  |
| 32 | *FXYD2* | *TERF2* |  |
| 33 | *CLEC5A* | *CCDC86* |  |
| 34 | *TMEM45B* | *CAB39L* |  |
| 35 | *SULT1B1* | *TST* |  |
| 36 | *UCHL1* | *OSTM1* |  |
| 37 | *SAMSN1* | *GNPDA1* |  |
| 38 | *DAPL1* | *GMNN* |  |
| 39 | *LAPTM5* | *FANCG* |  |
| 40 | *CBR3* | *B4GALT4* |  |
| 41 | *SLC7A11* | *PRIM2* |  |
| 42 | *PZP* | *TUBGCP5* |  |
| 43 | *PCOLCE2* | *MBNL3* |  |
| 44 | *MTHFD2* | *AZGP1* |  |
| 45 | *PCSK1N* | *HSPBAP1* |  |
| 46 | *SPP1* | *TPK1* |  |
| 47 |  | *AACS* |  |
| 48 |  | *GCNT1* |  |
| 49 |  | *UBE2D4* |  |
| 50 |  | *TMEM104* |  |
| 51 |  | *TLE4* |  |
| 52 |  | *RRAGD* |  |
| 53 |  | *TIMM10* |  |
| 54 |  | *F12* |  |
| 55 |  | *PER1* |  |
| 56 |  | *C1GALT1* |  |
| 57 |  | *MORC4* |  |
| 58 |  | *NINJ1* |  |
| 59 |  | *ARHGEF6* |  |
| 60 |  | *GNL3* |  |
| 61 |  | *LTF* |  |
| 62 |  | *SOX9* |  |
| 63 |  | *SACS* |  |
| 64 |  | *PTGER4* |  |
| 65 |  | *TMEM38B* |  |
| 66 |  | *ACP6* |  |
| 67 |  | *QPRT* |  |
| 68 |  | *ZNF576* |  |
| 69 |  | *PCDH9* |  |
| 70 |  | *CYP2U1* |  |
| 71 |  | *WASF3* |  |
| 72 |  | *CDR2L* |  |
| 73 |  | *DDB2* |  |
| 74 |  | *PDIA5* |  |
| 75 |  | *ACAT1* |  |
| 76 |  | *NEU1* |  |
| 77 |  | *ETFDH* |  |
| 78 |  | *PITPNM1* |  |
| 79 |  | *CDK2AP2* |  |
| 80 |  | *PPID* |  |
| 81 |  | *AKAP12* |  |
| 82 |  | *KCNJ15* |  |
| 83 |  | *SLC38A1* |  |
| 84 |  | *MCOLN3* |  |
| 85 |  | *GRAMD1B* |  |
| 86 |  | *KCNJ6* |  |
| 87 |  | *CRNKL1* |  |
| 88 |  | *GPX7* |  |
| 89 |  | *ARHGAP29* |  |
| 90 |  | *SLC31A2* |  |
| 91 |  | *KIF1C* |  |
| 92 |  | *KLF4* |  |
| 93 |  | *EDIL3* |  |
| 94 |  | *PLEKHO2* |  |
| 95 |  | *SEMA5A* |  |
| 96 |  | *PLAAT3* |  |
| 97 |  | *PON1* |  |
| 98 |  | *CLIC4* |  |
| 99 |  | *SMARCD3* |  |
| 100 |  | *CRP* |  |
| 101 |  | *MMACHC* |  |
| 102 |  | *CD14* |  |
| 103 |  | *C3AR1* |  |
| 104 |  | *CCR1* |  |
| 105 |  | *KCNAB2* |  |
| 106 |  | *BIRC3* |  |
| 107 |  | *MMP14* |  |
| 108 |  | *SRGN* |  |
| 109 |  | *ELOVL6* |  |
| 110 |  | *SOX4* |  |
| 111 |  | *ITPR3* |  |
| 112 |  | *BDH1* |  |
| 113 |  | *ASCL2* |  |
| 114 |  | *CCNJL* |  |
| 115 |  | *PHGDH* |  |
| 116 |  | *DGKD* |  |
| 117 |  | *NAALAD2* |  |
| 118 |  | *CSTA* |  |
| 119 |  | *ROR2* |  |
| 120 |  | *GAD1* |  |
| 121 |  | *TCN1* |  |
| 122 |  | *PPM1E* |  |
| 123 |  | *RASGRF1* |  |
| 124 |  | *CAPN6* |  |
| 125 |  | *ESM1* |  |
| 126 |  | *DDX25* |  |
| 127 |  | *IL18BP* |  |
| 128 |  | *CTSV* |  |
| 129 |  | *MS4A6A* |  |
| 130 |  | *DCX* |  |
| 131 |  | *ADCYAP1* |  |
| 132 |  | *RGS17* |  |

Table S2. COPD and T2DM expression profile data sets from GEO database (*mus musculus*)

| **Database**  **(COPD/T2D)** | **Dataset ID** | **Model** | **Platform** | **Number** | |
| --- | --- | --- | --- | --- | --- |
|  |  |  |  | **disease** | **controls** |
| **COPD** | GSE31950 | Transgenic(Tg) mice over-expressing TGFb1-Tg | GPL3677 | 4 | 4 |
| **COPD** | GSE52509 | CS-exposed WT mice | GPL6885 | 3 | 3 |
| **COPD** | GSE39304 | BALB/cJ mice treated with intranasally with poly I:C | GPL1261 | 6 | 6 |
| **COPD** | GSE38075 | IL-1b-Tg mouse COPD model | GPL6794 | 5 | 3 |
| **T2DM** | GSE123394 | C57BI/6（B6）mice induced by high fat diet | GPL81 | 4 | 4 |
| **T2DM** | GSE27213 | C57BL/6J mice induced by high-fat diet and HF-DR diet | GPL7202 | 12 | 12 |
| **T2DM** | GSE36032 | high fat diet induced mice | GPL11533 | 3 | 4 |
| **T2DM** | GSE197101 | NZO/HI mice | GPL6246 | 5 | 5 |

Table S3. The DEGs identified by Method1 in studies of *mus musculus*

| **NO** | **DEGs** |
| --- | --- |
| 1 | *pon1* |
| 2 | *cd14* |
| 3 | *slc16a6* |
| 4 | *il1rn* |
| 5 | *csf2rb2* |
| 6 | *mrc1* |
| 7 | *ccl6* |
| 8 | *spp1* |
| 9 | *tnfaip2* |
| 10 | *myo5a* |
| 11 | *aif1* |
| 12 | *ctss* |
| 13 | *slc39a4* |
| 14 | *havcr2* |
| 15 | *arl11* |
| 16 | *il1rl1* |
| 17 | *snx10* |
| 18 | *ctsk* |
| 19 | *trem2* |
| 20 | *gpnmb* |
| 21 | *npr3* |
| 22 | *adamts2* |
| 23 | *klrd1* |
| 24 | *ccl12* |
| 25 | *cotl1* |
| 26 | *ccr5* |
| 27 | *mmp12* |

Table S4. AUC of 6 hub genes

| **Gene** | **AUC of ROC** | **95% CI** |
| --- | --- | --- |
| *CCR1* | 0.8099 | 0.7856-0.8343 |
| *ITPR3* | 0.8090 | 0.7847-0.8332 |
| *AACS* | 0.8084 | 0.7839-0.8328 |
| *OPA1* | 0.7991 | 0.7742-0.8240 |
| *RGS17* | 0.7990 | 0.7740-0.8239 |
| *TUBGCP5* | 0.7893 | 0.7639-0.8148 |

Fig.S1 Weighted co-expression network analysis for identification and analyses of hub genes from the combined COPD datasets (Method3)

**A, B** β = 24 is selected as the soft threshold with the combined analysis of scale independence and average connectivity. **C** Clustering dendrogram of the COPD and control samples. **D** Gene co-expression modules represented by different colors under the gene tree. **E** Heatmap of eigengene adjacency. **F** Heatmap of the association between modules and COPD. The black, blue, magenta, red, and royalblue modules were significantly different (p < 0.1) and were considered as the interesting modules in COPD. Numbers at the top and bottom brackets represent the correlation coefficient and p-value, respectively. **G-J** Correlation plot between module membership and gene significance of genes included in the blue, royalblue, magenta, and black modules.

Fig.S2 Weighted co-expression network analysis for identification and analyses of hub genes from the combined T2DM datasets (Method3)

**A, B** β = 6 is selected as the soft threshold with the combined analysis of scale independence and average connectivity. **C** Clustering dendrogram of the T2DM and control samples. **D** Gene co-expression modules represented by different colors under the gene tree. **E** Heatmap of eigengene adjacency. **F** Heatmap of the association between modules and T2DM. The cyan, greenyellow, lightgreen, magenta, midnightblue, pink, purple, salmon, turquoise, and yellow modules were significantly different (p < 0.1) and were considered as the interesting modules in T2DM. Numbers at the top and bottom brackets represent the correlation coefficient and p-value, respectively. **G, H** Correlation plot between module membership and gene significance of genes included in the magenta and yellow modules.

Fig.S3 Schematic plot of the combination in *mus musculus*
